# Is Lipid Profile Associated with Bone Mineral Density and Bone Formation in Subjects with Spinal Cord Injury?

**DOI:** 10.1155/2014/695014

**Published:** 2014-08-21

**Authors:** Hadis Sabour, Abbas Norouzi Javidan, Sahar Latifi, Mohammad Reza Hadian, Seyed-Hassan Emami Razavi, Farzad Shidfar, Mohammad Reza Vafa, Hamidreza Aghaei Meybodi

**Affiliations:** ^1^Brain and Spinal Injury Research Center (BASIR), Tehran University of Medical Sciences, P.O. Box 6114185, Tehran, Iran; ^2^Nutrition Department, Iran University of Medical Sciences, Tehran, Iran; ^3^Endocrinology and Metabolism Research Institute (EMRI), Tehran University of Medical Sciences, Tehran, Iran

## Abstract

*Purpose*. The association between serum lipids and bone mineral density (BMD) has been investigated previously but, up to now, these relationships have not yet been described in spinal cord injury (SCI). We tried to assess the correlation between serum triglyceride (TG), total cholesterol (TC), high-density lipoprotein (HDL), and low-density lipoprotein (LDL) and BMD in male subjects with SCI.* Methods*. Dual-energy X-ray absorptiometry (DXA) was used to assess BMD in femoral neck, trochanter, intertrochanteric zone, and lumbar vertebras. Blood samples were taken to measure serums lipids and bone biomarkers including osteocalcin, cross-linked type I collagen (CTX), and bone alkaline phosphatase (BALP). Partial correlation analysis was used to evaluate the relationships between mentioned measurements after adjustment for weight and age.* Results*. We found a positive correlation between HDL and femoral neck BMD (*P*: 0.004, *r* = 0.33). HDL was negatively correlated with osteocalcin (*P*: 0.017, *r* = −0.31) which was not in consistency with its relationship with BMD. TC and LDL were not related to CTX, BALP and BMD. * Conclusion*. This study does not support a strong association between serum lipids and BMD in subjects with SCI. Moreover it seems that positive association between HDL and BMD is not mediated through increased bone formation.

## 1. Introduction

Existence of a probable relationship between lipid profile and bone mineral density (BMD) was initially derived from reported results on statins' positive effect on BMD [1, 2]. Various studies have supported the promising influence of statins (the HMG co-A reductase inhibitors) on reduction of fractures along with increased BMD [3, 4]. The assumption of relationship between lipids and BMD was tested in two cohort studies and showed a significant positive association between BMD and low density lipoprotein (LDL) and total triglyceride (TG) while it was negatively correlated with high density lipoprotein (HDL) [5]. However some other literatures showed no relationship between lipid profile and BMD [6]. Most of these studies were on healthy individuals. Spinal cord injury (SCI) is a severe medical condition which restricts physical activities tremendously [7]. Along with immobility, it is associated with some changes in lipid profile [8–10]. In this regard, de Groot et al. demonstrated decreased TC and elevated HDL during and one year after rehabilitation; however these patients may also experience increased levels of TC and LDL after discharge [8]. The study of de Groot also revealed the effect of age in subjects with SCI since higher TG, LDL, and HDL were observed in older patients. Wong et al. [9] reported the decreased level of HDL in subjects with SCI and their study also illustrated the effect of injury level since higher TG was detected among patients with injury at lumbar level. Moreover, osteoporosis occurs among these patients due to mechanical unloading [11–14] along with alterations in bone metabolism [15]. To our knowledge, the effect of serum lipids on BMD in subjects with SCI is still unknown and mostly the assessed population was postmenopausal women [16–18] which is a known population with higher risk of osteoporosis. In this study, we investigated only male subjects to omit the bias effect of menopause and other osteoporosis gender-related risk factors. Subjects with SCI also represent a sensitive population susceptible to osteoporosis and evaluation of the correlation between lipids and BMD in these patients is the main purpose of this study.

Changes in bone mass are associated with alterations in bone biomarkers. Lipids may affect BMD through their influence on bone biomarkers. In this field there are some specific known biomarkers including cross-linked type I collagen (CTX), osteocalcin, and bone alkaline phosphatase (BALP). CTX represents bone resorption and its increased level has been shown in SCI [19]. Osteocalcin and BALP are associated with bone formation. Our previous study showed that osteocalcin is negatively related to the BMD of femoral intertrochanteric zone [15]. We also demonstrated the positive association between CTX, BALP, and osteocalcin which shows the coincidental occurrence of osteoblastic and osteoclastic activities among subjects with SCI [15]. In the present study, we tried to assess correlations between bone biomarkers and lipid profile. Up to now, the association between BMD and lipid profile in subjects with SCI has not yet been reported and here in this study we tried to evaluate this relationship.

## 2. Materials and Methods

### 2.1. Study Design

This study was a cross-sectional investigation which was performed in Brain and Spinal Injury Research Center (BASIR). Data was collected from November 2010 to 2011. All patients received adequate information about the study and written consents were obtained before enrollment. The protocol was approved by the ethics committee at Tehran University of Medical Sciences.

### 2.2. Participants

Referred patients with SCI were invited to participate in this study. Inclusion criteria were male subjects, SCI with traumatic etiologies, and time since injury of more than 1 year. Exclusion criteria were pregnancy, lactation, amputation, recent fracture, and nontraumatic SCI etiology. Patients with history of diabetes, cancer, endocrinology disease, acute infection, coronary artery diseases, use of special medications such as glucocorticoid, hormones, thyroid hormones, anticonvulsive drugs, heparin, aluminum containing antacids, lithium, omega-3 fatty acids, or other nutrients supplements, and smoking or alcohol consumption were also excluded.

### 2.3. Anthropometric Measurements

Patients' demographic characteristics including age and postinjury duration were obtained during face-to-face interviews. Body weight was measured using a digital wheelchair scale, body height was obtained measuring the supine length, and body mass index (BMI) was calculated as body weight (in kilograms) divided by height (in meters) squared.

### 2.4. Bone Mineral Density Measurements

Dual-energy X-ray absorptiometry (DXA) was used to assess BMD in three bone sites of femur (neck, intertrochanteric zone, and trochanter) and spinal lumbar vertebras. Total hip BMD was measured as well. Calibration of bone densitometer Lunar DPXMD device (Lunar Corporation, Madison, WI, USA) was performed weekly by using appropriate phantoms. The precision error (PE) is usually expressed as the coefficient of variation (CV), which is the ratio of the SD to the mean of the measurements [20]. The precision error for bone mineral density measurements was 2 to 3% in the femoral and 1 to 1.5% in the lumbar regions. All scans were performed according to the manufacturer's guidelines. In patients with spinal implant the involved lumbar vertebras were excluded and the mean bone density of noninvolved vertebras was entered into the analysis.

### 2.5. Laboratory Measurements

Blood samples were taken under antiseptic conditions from antecubital vein. Blood samples were collected and centrifuged at 3000 rpm for 10 minutes at 4°C. Single session analysis was used to reduce interassay variation in serum samples. Samples were sent to the Endocrinology and Metabolism Research Center (EMRC) laboratory for analysis and were frozen immediately. Serums HDL, LDL, total cholesterol (TC), and total triglyceride (TG) were measured. Circulating levels of osteocalcin and C-telopeptide cross-linked type 1 collagen (CTX) were quantified by electrochemiluminescence immunoassay with detection limits of 0.50 ng/mL for osteocalcin and 0.07 ng/mL for CTX. BALP was measured by bone-specific alkaline phosphatase (BALP) ELISA kit with detection range of 1.25 ng/mL–80 ng/mL. All measurements were performed in Endocrinology and Metabolism Research Center (EMRC).

### 2.6. Statistical Analysis

All statistical analysis was performed by SPSS version 21 (IBM Corporation). The relationship between lipid profile indexes and anthropometric variables (age, weight, BMI, and time since injury) was assessed by simple linear regression. The association between BMD in different bone sites and lipids was evaluated using partial correlation with adjustment for weight, height, BMI, and age. *P* value < 0.05 was considered statistically significant.

## 3. Results

Total of 85 subjects with mean age of 51.80 ± 13.44 entered this investigation. Basic characteristics of these patients are summarized in Table 1. Eighteen subjects (21.2%) had injury at cervical level, 50 (58.8%) at thoracic level, and 17 (20%) at lumbar level. Mean TG was slightly higher among subjects with injury at lumbar level (170.29 ± 85 in lumbar group and 151.54 ± 58.97 and 138.55 ± 53 in thoracic and cervical groups, resp.,) but this difference was not statistically significant (*P*: 0.34). TC, HDL, and LDL also showed no relationship with injury level (*P*: 0.74, 0.68, and 0.71, resp.).

Mean triglyceride was 152.53 ± 64.06 and mean of TC was 175.69 ± 34.38. Means of HDL and LDL were 41.31 ± 8.94 and 100.49 ± 24.97, respectively.

The primary analysis of confounders effects including age, weight, height, and BMI on serum lipids showed that there is a significant negative association between weight and HDL (*P*: 0.002, *r* = −0.32) and also between age and TC as well as LDL (*P* < 0.0001, *r* = −0.38 and *P* < 0.0001, *r* = −0.36, resp.). Weight was positively related to TG (*P*: 0.003, *r* = 0.31). BMI was correlated with LDL (*P*: 0.024, *r* = 0.24), TG (*P*: 0.007, *r* = 0.29), and HDL (*P*: 0.009, *r* = −0.28). The existence of these statistically significant relationships defines these mentioned factors as confounder [21], so, in the next step of analysis, the relationship between serum lipids and BMD and bone biomarkers was evaluated after adjustment for mentioned factors.

After adjustment for age, weight, height, and BMI, the correlation between serum lipids and BMD was assessed. The effect of lipids on bone biomarkers was evaluated as well. We found a positive correlation only between HDL and femoral neck BMD (Table 2). Furthermore, we did not find any significant effect of serum lipids on CTX and BALP (Table 2) while HDL was negatively correlated with osteocalcin. The results are interesting as the positive effect of HDL on femoral neck BMD is not consistent with negative association between osteocalcin and HDL because mostly we consider osteocalcin as a bone formation indicator. These results show that serum lipids and especially HDL may affect long bone BMD in patients with SCI through mechanisms which are not merely mediated by pathways in which bone biomarkers' alterations are involved.

## 4. Discussion

The probable association between serum lipids and BMD has been proposed when the positive effect of lipid reducing medications such as statins in increasing BMD was observed in various studies [22, 23]. However, while Wada et al. [24] detected no association between statin use and lumbar BMD, other literatures have shown that reduced fracture risk is greater than statin-induced increased BMD [25]. These correlations seem to be more evident in patients with increased risk of osteoporosis rather than healthy able-bodies [16, 17, 26]. The hypothesis of the association between atherogenic lipid profiles and BMD has been tested in several studies and the controversial results have been published up to now. Some investigations supported the negative association between atherogenic lipid profile and BMD [18], some found no relationship [6, 27], and some studies reported positive correlation [5, 17]. Solomon et al. [6] investigated a large number of able-bodies in a national survey and found no association between serum lipids and BMD. This study is noticeable by considering its sample size. However, these relationships may be different in individuals with background diseases or medical conditions that are known to be associated with osteoporosis. Here, in male subjects with SCI, we found that femoral neck BMD was positively related to HDL. Previously Dennison et al. [28] showed a negative correlation between HDL and femoral BMD in healthy individuals which contradicts with our results in subjects with SCI. The results of de Groot et al. [8] showed that, after SCI, an elevated level of TC and LDL can be observed which can be reduced during rehabilitation. This unfavorable hyperlipidemia that occurs after SCI may play a part in the controversial results between our study and Dennison's investigation on healthy subjects. Wong et al. [9] illustrated the effect of injury level on lipid profile and their study showed higher level of TC among patients with injury at lumbar level. We found no significant relationship between injury level and TC which contradicts with Wong's study. Many various factors such as ethnicity, diets, and life-style affect lipid profile and may result in such contradictions between our results in Iranian patients and Wong's study in Chinese population. It has been shown that lipid profile in subjects with SCI goes under some alterations after SCI which is mostly toward reduction of HDL in these patients [29–31]. The negative correlation between HDL and BMD which was reported in mentioned studies does not lead to increased BMD in subjects with SCI because bone loss in these patients is a consequence of various factors including mechanical unloading and changes in bone metabolism. As a result we do not observe such a negative association between HDL and BMD in subjects with SCI. The controversial results in different study populations suggest that lipids' relationship with BMD is manipulated by many various factors and, apart from background medical condition, factors like lifestyle, physical activity, and amount of fat mass should be considered.

Most studies found no association between TC and BMD in various populations [27, 32, 33] which is in agreement with our results in SCI. However, while Parhami et al. [34] illustrated that baseline level of cholesterol synthesis is necessary for the osteoblastic differentiation, it seems that total cholesterol does not directly affect BMD. Here, we report the same findings in subjects with SCI.

CTX which represents bone resorption and whose negative association with BMD has been frequently demonstrated [35, 36] does not seem to be related to serum lipids. BALP which is a factor reflecting bone formation did not show any relationship with lipids. However, osteocalcin which is a specific marker illustrating bone formation and osteoblastic activity [37] was negatively associated with HDL in men. These results show that positive association between HDL and BMD in male subjects with SCI is not mediated through pathways which involve increased osteocalcin. In fact this result suggests that HDL positive relationship with BMD does not affect bone formation markers, which proposes that this effect is not mediated through increased bone formation.

LDL was also not related to BMD in subjects with SCI, which is in agreement with Dennison's report in general population [28]. Many studies do not support strong association between lipids' concentrations and bone mass measurements [38]. In this study, we report also a weak relationship between lipid levels and BMD in SCI population. Only HDL was positively associated with femoral neck BMD. LDL and TC levels revealed no influence on BMD.

## 5. Conclusion

This study does not support a strong association between serum lipids and BMD in patients with SCI. LDL and TC had no relationship with BMD in male subjects with SCI while HDL was positively associated with femoral neck BMD. This positive correlation between HDL and BMD was not in line with bone biomarkers' alterations as osteocalcin, which is a bone formation marker, was negatively associated with HDL and no other relationship with CTX and BALP was observed. These results suggest that the relationship between serum lipids and BMD in subjects with SCI is weak. This study shows a positive correlation between HDL and femoral neck bone mineral density (BMD) and a negative association between HDL and osteocalcin in male subjects with spinal cord injury (SCI). TC and LDL were not related to BMD and bone biomarkers in SCI.

## Figures and Tables

**Table 1:** Baseline characteristics of study cases and differences of basic features among male subjects with spinal cord injury (*n*: 85).

Category		Mean (standard deviation)
Age (year)		51.80 (13.44)

Weight (kg)		70.49 (14.36)

Height (cm)		172.34 (6.92)

Body mass index (kg/m^2^)		23.62 (4.05)

Time since injury (year)		9.15 (6.43)

Triglyceride (mg/dL)		152.54 (64.06)

Total cholesterol (mg/dL)		175.69 (34.38)

High density lipoprotein (mg/dL)		41.31 (8.94)

Low density lipoprotein (mg/dL)		100.49 (24.97)

Bone mineral density of femoral neck	*T*-score	−2.16 (0.95)
	*Z*-score	−1.74 (0.96)

Bone mineral density of femoral intertrochanteric zone	*T*-score	−2.29 (0.94)
	*Z*-score	−2.21 (0.95)

Bone mineral density of femoral trochanter	*T*-score	−2.11 (0.73)
	*Z*-score	−1.92 (0.74)

Bone mineral density of total hip	*T*-score	−2.36 (0.83)
	*Z*-score	−2.22 (0.86)

Bone mineral density of spinal lumbar vertebras	*T*-score	−0.43 (1.81)
	*Z*-score	0.53 (1.59)

**Table 2:** The relationship between serum lipids and bone mineral density in male subjects with spinal cord injury.

Category		Male subjects with spinal cord injury (*n*: 85)			
		TG	TC	HDL	LDL
Femoral neck BMD	*T*-score	0.17	0.84	0.004∗∗ (*r* = 0.33)	0.42
	*Z*-score	0.18	0.81	0.004∗∗ (*r* = 0.35)	0.45

Femoral intertrochanteric zone BMD	*T*-score	0.28	0.49	0.08	0.90
	*Z*-score	0.29	0.45	0.07	0.86

Femoral trochanter BMD	*T*-score	0.88	0.73	0.97	0.95
	*Z*-score	0.87	0.71	0.90	0.94

Total hip BMD	*T*-score	0.36	0.42	0.09	0.85
	*Z*-score	0.40	0.41	0.09	0.85

Spinal lumbar vertebras' BMD	*T*-score	0.56	0.52	0.78	0.27
	*Z*-score	0.73	0.74	0.31	0.95

Osteocalcin		0.99	0.22	0.017∗ (*r* = −0.31)	0.91

Cross-linked type I collagen		0.56	0.79	0.64	0.97

Bone alkaline phosphatase		0.76	0.65	0.92	0.40

BMD: bone mineral density, HDL: high density lipoprotein, LDL: low density lipoprotein, TC: total cholesterol, and TG: total triglyceride.

∗Significance at level of *P* < 0.05.

∗∗Significance at level of *P* < 0.01.

## Abbreviations

BALP: Bone alkaline phosphatase
BMD: Bone mineral density
BMI: Body mass index
CTX: Cross-linked type I collagen
DXA: Dual-energy X-ray absorptiometry
HDL: High density lipoprotein
LDL: Low density lipoprotein
SCI: Spinal cord injury
TC: Total cholesterol
TG: Triglyceride.
